# Human leucocyte antigen class I and II alleles are associated with tumor susceptibility in neurofibromatosis type 1

**DOI:** 10.3389/fimmu.2026.1966259

**Published:** 2026-10-19

**Authors:** Jonathan H. Sussman, Stephanie N. Brosius, Bernat Gel, Peiyao Li, Alvin Farrel, Jo Lynne Rokita, Eduard Serra, Kai Tan, Michael J. Fisher, John M. Maris, Thomas De Raedt

**Affiliations:** 1 Department of Pediatrics, Division of Oncology, Children’s Hospital of Philadelphia, Philadelphia, PA, United States; 2 Department of Pediatrics, University of Pennsylvania Perelman School of Medicine, Philadelphia, PA, United States; 3 Graduate Group in Genomics and Computational Biology, Perelman School of Medicine, University of Pennsylvania, Philadelphia, PA, United States; 4 Medical Scientist Training Program, Perelman School of Medicine, University of Pennsylvania, Philadelphia, PA, United States; 5 Departament de Fonaments Clínics, Facultat de Medicina i Ciències de la Salut, Universitat de Barcelona (UB), Barcelona, Spain; 6 Centro de Investigación Biomédica en Red de Cáncer (CIBERONC), Madrid, Spain; 7 Center for Cancer and Immunology Research, Children’s National Hospital, Washington, DC, United States; 8 Department of Pediatrics, The George Washington University School of Medicine and Health Sciences, Washington, DC, United States; 9 Translational Genomics and Bioinformatics of Cancer, Germans Trias i Pujol Research Institute (IGTP), Badalona, Barcelona, Spain; 10 Hereditary Cancer Group, Germans Trias i Pujol Research Institute (IGTP), Badalona, Barcelona, Spain

**Keywords:** glioblastoma, high-grade glioma, HLA, low-grade glioma, MPNST, neurofibromatosis type 1, NF1

## Abstract

**Introduction:**

Neurofibromatosis type 1 (NF1) is a common autosomal dominant genetic tumor predisposition syndrome. NF1 patients display remarkable phenotypic variability, even within families carrying the same *NF1* mutation. With few exceptions, the identification of specific genotype-phenotype correlations has remained elusive.

**Methods:**

We utilized RNA-sequencing, whole exome sequencing data and direct DNA sequencing to determine HLA genotypes for individuals with NF1-associated high-grade glioma (HGG, n=38), low-grade glioma (LGG, n=91), and malignant peripheral nerve sheath tumors (MPNST, n=105). Odds ratios (OR), binomial p-values and false discovery values were calculated by comparing observed carrier frequencies against expected frequencies derived from ethnicity-matched population data.

**Results:**

We find that specific HLA class I and II alleles are associated with different NF1 tumor types. For example, HLA-B*40:02 is significantly associated with NF1-MPNST (OR=3.71, p=0.001, Q=0.023), increasing the lifetime risks for MPNST from 10-15.8% to about 36.6% (CI 16.1-57.2%).

**Discussion:**

The relative cancer risk for an individual in the general population carrying a risk allele can be high, however, that individual's absolute risk for cancer typically remains very low. In contrast, individuals that carry a risk allele and are also burdened with a tumor predisposition syndrome will have a substantially higher absolute risk to develop a tumor, simply because they start at a higher baseline susceptibility for tumors. The identification of HLA-risk alleles for NF1 tumor development has the potential to improve clinical care and outcomes by prompting risk-adapted screening and treatment. It is important to note that the associations found in this study require validation in an independent prospective cohort.

## 1 Introduction

Neurofibromatosis type 1 is a complex disorder with both tumor and non-tumor manifestations. NF1 is one of the most frequent genetic disorders affecting about 1 in 3000 individuals (1). Although it is a multi-system disease, as a tumor predisposition syndrome, patients are at particular risk to develop benign neurofibromas, low-grade glioma (LGG, including both optic pathway glioma and non-optic pathway glioma), malignant peripheral nerve sheath tumors (MPNST, 10-15.8% life-time risk) (2, 3) and high-grade glioma (HGG, 1-2% lifetime risk), which include glioblastoma (GBM) and high-grade astrocytoma with piloid features (HGAP) (1, 4, 5). It is important to note that manifestations of NF1 are very heterogeneous, even within members of the same family (6).

Individuals with NF1 have a pathogenic germline variant in, or deletion of, the *NF1* tumor suppressor, which is a negative regulator of the RAS pathway. Somatic loss of the wild-type copy of *NF1* can lead to tumor formation and forms the basis of many of the features present in NF1 patients. Few recurrent germline variants in *NF1* exist, making genotype-phenotype correlations challenging. Specific germline variations in *NF1* do not explain the full heterogeneity of clinical manifestations observed in NF1 (6, 7). With few exceptions, the identification of specific genotype-phenotype correlations has remained elusive (8–11). One of the rare examples of genotype-phenotype correlation is the increased risk for dermal neurofibromas and MPNST in those individuals with an *NF1* microdeletion (5%) (12). The 1.4 Mb NF1 microdeletion encompasses *NF1* and surrounding areas, including the tumor suppressor *SUZ12* which is often mutated in MPNST (13, 14). Specific germline pathogenic variants in *NF1* have been associated with large superficial plexiform and spinal neurofibromas (missense mutations in AA 844-848, which occur in 0.8% of NF1 individuals) (9); others are associated with a Noonan-like phenotype (p.Met1149, p.Arg1276 and p.Lys1423, which represent about 1.8% of unrelated NF1 individuals) (11). The p.Arg1276 pathogenic missense variants are associated with spinal neurofibromas; p.MET1149 individuals have a mild phenotype (11). It is important to note that collectively these alterations represent a small fraction of the NF1 population and do not explain the intra-familial variation of clinical manifestations.

Human leukocyte antigen (HLA) class I molecules, which include HLA-A, HLA-B and HLA-C exhibit remarkable genetic diversity across human populations. This diversity is driven by the need to recognize and present a broad range of peptides (antigens) from intracellular pathogens, such as viruses, or neoantigens from mutated genes to CD8 T cells (15). The 3 classic HLA class I genes are located at the same genomic region (Chromosome 6p22.1) and each has multiple alleles. These alleles encode a slightly different peptide-binding groove, allowing for a wide range of antigenic peptides to be presented (15). It is noteworthy that all cells continuously interact with the immune system by displaying processed peptides on HLA receptors. These peptides can be derived from viral or mutated proteins but are most frequently from normal proteins that are expressed in the cell. This HLA display system is used by the immune system to recognize cells that are infected or unhealthy (15). The diversity of alleles for each HLA gene enhances the flexibility and adaptability of the immune system, but also influences susceptibility to diseases, including infections, autoimmune disorders, and cancer (16). HLA class II molecules on the other hand are typically expressed in antigen presenting immune cells, and play an important role in immune cell activation, including CD4 T-cells. Intriguingly, it has become evident that over 50% of NF1-associated HGG tumor cells express these genes associated with HLA-class II antigen presentation that are typically restricted to immune cells (17). Expression of HLA-class II genes on non-immune cells is generally thought to be immune suppressive. We therefore investigated if we would identify alleles from HLA-DRB1, the highest expressed HLA-class II gene, which are associated with or underrepresented in specific NF1 tumors. In this study, we identified associations between alleles of both HLA class I and II genes and the occurrence of LGG and MPNST in individuals with NF1. These observations will need to be validated in an independent prospective cohort of NF1 individuals.

## 2 Materials and methods

### 2.1 NF1 individuals

We used published and publicly available data (RNA-sequencing (RNA-seq), whole exome and/or whole genome sequencing) and/or materials deposited in the Children’s Brain Tumor Network (CBTN) biobank (DNA) to HLA-type individuals with NF1: 38 NF1-HGG patients (18, 19) (**Supplementary Table 1**), 91 NF1-LGG patients (18–21) (**Supplementary Table 2**) and 105 NF1-MPNST patients (19, 22–26) (**Supplementary Table 3**). For some patients the race/ethnicity has been reported (CBTN), but for other patients the genetic ancestry was determined using whole genome or whole exome sequencing when available. For most of these individuals the only material/data available was derived from tumors. The CBTN obtained informed consent from the patients (or their guardian) for samples obtained from the CBTN.

### 2.2 Non-NF1 individuals

We collected publicly available data (RNA-seq, CBTN (19)) of sporadic (non-tumor predisposition associated) counterparts of the NF1-associated tumors and included only those individuals in our study for whom reported race/ethnicity data is available: 204 pediatric HGG patients (pHGG; **Supplementary Table 4**) and 218 pediatric LGG patients (pLGG; **Supplementary Table 5**). We realize that both the pHGG and pLGG cohorts are heterogeneous and contain different subtypes of glioma, which could affect our analysis. However, by comparing NF1-associated gliomas with sporadic gliomas, we are looking for HLA alleles that are broadly underrepresented or associated with glioma formation.

### 2.3 HLA-typing

We HLA typed individuals with NF1 who developed HGG by using RNA-seq data analyzed with the arcasHLA algorithm (27) v0.6.0 (default parameters), by direct DNA sequencing, or by whole exome sequencing (WES) (**Supplementary Tables 1**–**5**). For WES, we used OptiType (28) v1.3.1 for HLA class I and HLA-HD (29) v1.7.1 for HLA class II both as implemented in the nf-core/hlatyping pipeline (30) v2.2.0 with default parameters against the default HLA-HD reference dictionary. We analyzed the alleles of the following HLA-genes in this study: HLA-A, HLA-B, HLA-C, HLA-DRB1. When HLA-genes were directly sequenced, we used PHLAT to identify specific alleles.

### 2.4 Race/ethnicity

Because the HLA frequencies vary across different populations, we determined the race/ethnicity of patients (White, Black, Asian, Hispanic). For some patients the race/ethnicity has been self-reported (CBTN), but for other patients the genetic ancestry was determined using whole genome or whole exome sequencing (when available) with Somalier (31) v0.3.1 using the ancestry module against the pre-calculated 1000 Genomes (1kg) dataset with default settings (**Supplementary Tables 1**–**5**).

### 2.5 Statistics

We determined the observed and expected percentage of patients (based on race/ethnicity HLA allele frequency data of patients at CHOP) that carry a specific HLA allele, calculated the odds ratio (OR) with confidence interval (CI), and two-sided binomial p-value. Odds ratios for each HLA allele were calculated by comparing the observed odds of allele carriage among cases to the odds implied by the ethnicity-matched expected population carrier frequency. The 95% confidence intervals for each odds ratio were calculated using the Copper-Pearson interval for the observed carrier proportion among cases, holding the expected population frequency fixed. These proportion bounds were converted to odds and divided by the fixed reference odds to yield the corresponding OR confidence bounds. Lifetime tumor risk for allele carriers was estimated using a Bayesian odds-scaling approach. To generate a conservative range that propagates uncertainty from both the odds ratio and the baseline tumor risk estimate, we paired the lower bound of the OR 95% CI with the lower bound of the published baseline risk range, and the upper bound of the OR 95% CI with the upper bound for the baseline risk range. This range represents a conservative bound on the possible lifetime risk rather than a formal joint confidence interval, since it combines the extremes of two independently estimated quantities.

For the binomial p-values, we also calculated the false discovery (Q) value using the Benjamini-Hochberg method for those alleles where at least 4 cases were observed or expected (NF1-HGG and NF1-LGG) or alleles with an observed or expected carrier frequency of at least 5% (MPNST, sporadic pediatric HGG, sporadic pediatric LGG). Unadjusted p-values and odds ratios are reported for all evaluated alleles. HLA alleles with a false discovery rate lower than 10%, were considered significant. Alleles with a Q-value between 10%-25% were considered borderline significant and considered exploratory signals given the small size of the cohorts (**Tables 1**–**5**; **Supplementary Table 7**). To evaluate associations between HLA allele carrier status and clinical features (e.g. age at diagnosis), two-tailed Fisher’s exact tests were performed on 2x2 contingency tables comparing binary clinical characteristics between carriers and non-carriers of specific alleles within the cohort subset with available clinical data.

**Table 1:** HLA-I and II associations of alleles significant in NF1 and sporadic cohorts in NF1-LGG.

NF1-LGG	B07:02	B13:02	B38:01	B40:02	C07:02	C14:02	DRB1-01:01	DRB1-04:03	DRB1-12:01	DRB1-16:01
cases (out of 91)	10	3	6	1	11	7	11	5	9	2
Patient carrier %	10.87	3.26	6.52	1.09	11.96	7.61	13.10	5.95	10.71	2.38
Expected carrier %	20.81	4.78	2.43	3.55	24.90	3.03	15.35	1.75	3.15	1.88
Odds Ratio	0.46	0.67	2.80	0.30	0.41	2.63	0.83	3.55	3.69	1.28
CI lower	0.22	0.14	1.01	0.01	0.20	1.04	0.40	1.12	1.62	0.15
CI upper	0.91	2.05	6.43	1.73	0.78	5.73	1.58	8.65	7.38	4.76
p-value	0.020	0.803	0.024	0.384	0.003	0.021	0.652	0.016	0.001	0.673
Q-value	0.196	NC	0.196	NC	0.074	0.196	0.990	0.196	0.049	NC
Association	NEG	NC	POS	NC	NEG	POS	NS	POS	POS	NC

CI, Confidence Interval; p-value, binomial p-value; Q-value, false discovery; NC, not calculated; POS, positive association; NEG, underrepresentation; NS, not significant. HLA-DRB1 data was available for 84 samples. Green, underrepresentation; Orange, overrepresentation; lighter shading denotes borderline significance.

## 3 Results

As part of an immunotherapy research program at the Children’s Hospital of Philadelphia (CHOP), we HLA typed individuals with NF1 who developed HGG and observed an overrepresentation of some HLA alleles. Intrigued by these observations, we expanded our NF1-HGG cohort and investigated new cohorts of NF1-LGG and NF1-MPNST as well as sporadic pHGG and sporadic pLGG (**Supplementary Tables 1**–**5**). Because HLA allele frequencies vary across race/ethnicity, we used reported ethnicity or determined their genetic ancestry from available whole genome or exome sequencing. This allowed us to determine the genetic ancestry of 89% of patients with NF1-HGG patients, 76% of patients with NF1-LGG patients and 67% of patients with NF1-MPNST. In the sporadic cohorts we only used individuals with self-reported ethnicity/race data. In all cohorts we observed a strong bias towards White ethnicity. We combined the self-reported and determined genetic ancestry to infer the expected frequencies of all HLA alleles in the entire cohort of each tumor type.

Because we analyzed sequencing data derived tumor material, we cannot exclude that loss of heterozygosity (LOH) of chromosome 6 (where the HLA genes are located) reduced the number of alleles identified in a patient. This could result in a slight undercounting of carriers of specific HLA alleles, which could lead to false positive identification of underrepresented alleles but simultaneously undercounting of alleles associated with tumor formation. MPNST and HGG, which are more prone to copy number variations (CNV) and LOH, have the highest risk of introducing this bias. LOH events are rare in NF1-associated LGG, reducing our concern for biases in that cohort. Moreover, only MPNST, and not NF1-HGG nor NF1-LGG showed loss of Hardy-Weinberg equilibrium (**Supplementary Table 6**) of the HLA gene loci, suggesting some LOH took place in some MPNST tumors. Crucially, nearly all samples showed heterozygous HLA alleles across HLA-A, -B and/or C (37/38 NF1-HGG, 94/105 MPNST and 90/91 NF1-LGG), reducing our concern of an LOH bias in our analysis. For patients with both RNA-seq and WES (d’Angelo cohort HGG/LGG, n=27 with tumor WES, n=20 with germline WES), we found strong overall concordance: 209/216 (97%) alleles were concordant between RNA-seq and tumor WES and 157/160 (98%) alleles were concordant between RNA-seq and germline WES. We also directly compared the samples that had both tumor and germline WES from that dataset (n=44) and found almost perfect concordance with 351/352 (99.7%) alleles matching. This indicates that HLA-typing with RNA-seq on tumor tissue is accurate and that the error introduced by tumor-derived genomic alterations in glioma is negligible.

### 3.1 HLA-class I associations in NF1-LGG

We find that HLA-class I HLA-C*07:02 is significantly depleted in our NF1-LGG cohort, meeting our false discovery rate cutoff (Q<0.1) with an OR = 0.41 (CI = 0.20-0.78), binomial p-value=0.003 and Q = 0.074 (**Table 1**). It is not significantly depleted or enriched in the NF1 associated HGG or MPNST (**Tables 2**, **3**) nor in sporadic pLGG or pHGG cohorts (**Tables 4**, **5**). A second allele, HLA-B*07:02 was underrepresented with borderline significance (OR = 0.46, CI = 0.22-0.91, p=0.020, Q = 0.20) in the NF1-LGG cohort. These two alleles (HLA-C*07:02 and HLA-B*07:02) are in near-complete linkage disequilibrium in European ancestry populations (32), indicating that this depletion likely represents a single haplotype. Interestingly, the HLA-C*07-B*07 haplotype has been previously found to be positively associated with glioblastoma risk (33), which is in contrast to the protective association that we observe in NF1-LGG patients. Two other alleles (HLA-B*38:01 and HLA-C*14:02) were positively associated with borderline significance in NF1-LGG with Q values between 0.1 and 0.25 (**Table 1**).

**Table 2:** HLA-I and II associations of alleles significant in NF1 and sporadic cohorts in NF1-HGG.

NF1-HGG	B07:02	B13:02	B38:01	B40:02	C07:02	C14:02	DRB1-01:01	DRB1-04:03	DRB1-12:01	DRB1-16:01
cases (out of 38)	6	5	0	2	6	3	4	1	2	1
Patient carrier %	15.79	13.16	0.00	5.26	15.79	7.89	10.81	2.70	5.41	2.70
Expected carrier %	21.43	4.93	2.23	3.15	25.03	3.04	15.85	1.61	3.19	1.90
Odds Ratio	0.69	2.92	0.00	1.71	0.56	2.73	0.64	1.70	1.73	1.43
CI lower	0.24	0.89	0.00	0.20	0.19	0.54	0.17	0.04	0.20	0.04
CI upper	1.67	7.53	4.48	6.62	1.36	8.67	1.81	10.07	6.75	8.51
p-value	0.552	0.038	1.000	0.338	0.260	0.108	0.505	0.452	0.331	0.509
Q-value	0.764	0.586	NC	NC	0.657	NC	0.764	NC	NC	NC
Association	NS	NS	NC	NC	NS	NC	NS	NC	NC	NC

CI, Confidence Interval; p-value, binomial p-value; Q-value, false discovery; NC, not calculated; POS, positive association; NEG, underrepresentation; NS, not significant. HLA-DRB1 data was available for 37 samples. Green, underrepresentation; Orange, overrepresentation; lighter shading denotes borderline significance.

**Table 3:** HLA-I and II associations of alleles significant in NF1 and sporadic cohorts in NF1-MPNST.

NF1-MPNST	B07:02	B13:02	B38:01	B40:02	C07:02	C14:02	DRB1-01:01	DRB1-04:03	DRB1-12:01	DRB1-16:01
cases (out of 105)	15	4	4	10	18	3	4	2	1	1
Patient carrier %	14.29	3.81	3.81	9.52	17.14	2.86	4.17	2.08	1.04	1.04
Expected carrier %	20.78	4.74	2.23	2.76	24.36	3.26	15.05	1.61	3.66	1.73
Odds Ratio	0.64	0.80	1.74	3.71	0.64	0.87	0.25	1.30	0.28	0.60
CI lower	0.34	0.21	0.46	1.72	0.36	0.18	0.06	0.14	0.01	0.01
CI upper	1.10	2.10	4.59	7.13	1.08	2.62	0.59	4.39	1.44	3.12
p-value	0.117	0.820	0.301	0.001	0.089	1.000	0.001	0.688	0.191	1.000
Q-value	0.519	NC	NC	0.023	0.519	NC	0.023	NC	NC	NC
Association	NS	NC	NC	POS	NS	NC	NEG	NC	NC	NC

CI, Confidence Interval; p-value, binomial p-value; Q-value, false discovery; NC, not calculated; POS, positive association; NEG, underrepresentation; NS, not significant. HLA-DRB1 data was available for 96 samples. Green, underrepresentation; Orange, overrepresentation; lighter shading denotes borderline significance.

**Table 4:** HLA-I and II associations of alleles significant in NF1 and sporadic cohorts in sporadic pediatric HGG.

Sporadic pHGG	B07:02	B13:02	B38:01	B40:02	C07:02	C14:02	DRB1-01:01	DRB1-04:03	DRB1-12:01	DRB1-16:01
cases (out of 204)	37	11	9	7	53	10	24	5	12	9
Patient carrier %	18.14	5.39	4.41	3.43	25.98	4.90	11.76	2.45	5.88	4.41
Expected carrier %	19.41	4.46	2.79	4.05	24.44	3.16	14.13	2.02	3.29	1.75
Odds Ratio	0.92	1.22	1.61	0.84	1.08	1.58	0.81	1.22	1.84	2.59
CI lower	0.63	0.60	0.72	0.33	0.78	0.75	0.51	0.39	0.93	1.17
CI upper	1.32	2.23	3.12	1.77	1.49	2.97	1.24	2.89	3.29	5.02
p-value	0.723	0.495	0.194	0.858	0.625	0.157	0.367	0.613	0.047	0.010
Q-value	0.953	0.953	NC	NC	0.953	NC	0.920	NC	0.620	NC
Association	NS	NS	NC	NC	NS	NC	NS	NC	NS	POS

CI, Confidence Interval; p-value, binomial p-value; Q-value, false discovery; NC, not calculated; POS, positive association; NEG, underrepresentation; NS, not significant. Green, underrepresentation; Orange, overrepresentation; lighter shading denotes borderline significance.

**Table 5:** HLA-I and II associations of alleles significant in NF1 and sporadic cohorts in sporadic pediatric LGG.

Sporadic pLGG	B07:02	B13:02	B38:01	B40:02	C07:02	C14:02	DRB1-01:01	DRB1-04:03	DRB1-12:01	DRB1-16:01
cases (out of 218)	37	21	7	11	48	9	27	10	7	15
Patient carrier %	16.97	9.63	3.21	5.05	22.02	4.13	12.39	4.59	3.21	6.88
Expected carrier %	20.99	4.66	2.28	3.31	24.28	2.86	15.19	1.49	3.33	1.85
Odds Ratio	0.77	2.18	1.42	1.55	0.88	1.46	0.79	3.17	0.96	3.93
CI lower	0.52	1.32	0.57	0.76	0.63	0.66	0.51	1.50	0.38	2.16
CI upper	1.1	3.43	2.98	2.84	1.22	2.83	1.19	5.95	2.02	6.63
p-value	0.158	0.002	0.356	0.179	0.477	0.225	0.299	0.002	1.000	0.000
Q-value	0.441	0.049	NC	0.441	0.696	NC	0.588	NC	NC	0.000
Association	NS	POS	NC	NS	NS	NC	NS	NC	NC	POS

CI, Confidence Interval; p-value, binomial p-value; Q-value, false discovery; NC, not calculated; POS, positive association; NEG, underrepresentation; NS, not significant. Green, underrepresentation; Orange, overrepresentation; lighter shading denotes borderline significance.

### 3.2 HLA-class I associations in NF1-HGG

The NF1-HGG cohort is the smallest in the dataset (n=38 cases) given its lifetime risk of 1-2% in NF1 patients. We did not find any significant or exploratory associations with NF1-HGG in this cohort (Q<0.25), nor did we identify any significant associations (Q<0.25) in the sporadic NF1-HGG cohort of 204 individuals. One allele (HLA-B13:02) had a nominal p-value of 0.038 for an increased association (OR = 2.92, CI = 0.89-7.53) but did not pass multiple hypothesis testing (Q = 0.59) (**Table 2**). However, this allele was significantly associated with an increased risk for sporadic pLGG (OR = 2.18, CI = 1.32-3.43, p=0.002, Q = 0.049) but not sporadic pHGG nor NF1-LGG.

### 3.3 HLA-class I associations in NF1-MPNST

One HLA-class I allele, HLA-B*40:02, is carried in 10 of 105 individuals with NF1-MPNST (9.5%), while we expect it in only 2.8% of individuals (OR = 3.71, CI = 1.72-7.13; p=0.001, binomial test, Q = 0.023) (**Table 3**). The allele frequency of HLA-B*40:02 is uniformly low across races (Caucasian 1.3%, African American 0.3%, Hispanic 4.9%, Asian 2.4%). This association between HLA-B*40:02 and NF1-MPNST means that carriers of HLA-B*40:02 have an elevated estimated lifetime risk for MPNST of 36.6% (CI = 16.0-57.2%), a risk that was calculated using Bayes’ theorem (see Methods), which is higher than the expected 10%-15.8% lifetime risk (2, 3) for MPNST in the general NF1 population. This lifetime risk estimate accounts for uncertainty in both the baseline lifetime risk and the odds ratio confidence interval. Strikingly, this allele is especially frequent in NF1 individuals who developed an MPNST before the age of 18. Of the 9 pediatric patients in our NF1-MPNST cohort, 4 carry HLA-B*40:02, which is significantly higher than the number of pediatric MPNST patients among individuals with NF1 that do not carry HLA-B*40:02 (p=0.0027, Fisher exact test, Age of diagnosis data was available for 34/105 MPNST patients). The MPNST study included patients from several hospitals/institutes reducing the risk of ascertainment bias on age (23–26).

### 3.4 HLA-class II gene DRB1 associations in NF1 tumors

We performed a similar analysis for HLA-class II gene DRB1, which is typically only expressed on professional antigen presenting immune cells. However, some NF1 tumors express components of the HLA-class II antigen presentation machinery (17). We identified 2 alleles that were significantly associated with or underrepresented in NF1-associated tumors, namely HLA-DRB1*12:01 in NF1-LGG and HLA-DRB1*01:01 in MPNST respectively (**Tables 1**–**3**). Noteworthy is HLA-DRB1*12:01, which is associated with NF1-LGG (OR = 3.69, CI = 1.62-7.38, p=0.001, Q = 0.049) but is not enriched in the sporadic pediatric LGG or HGG populations nor NF1-HGG and NF1-MPNST. Additionally, we identified HLA-DRB1*01:01, which is significantly underrepresented in NF1-MPNST patients (OR = 0.25, CI = 0.06-0.59, p<0.001, Q = 0.046), suggesting this is a protective allele in MPNST. We found that HLA-DRB1*04:03 was borderline significant in the NF1-LGG cohort (OR = 3.55, CI = 1.12-8.65, p=0.016, Q = 0.20). This same allele a nominal p-value of 0.002 in the larger sporadic pLGG cohort with a similar odds ratio (OR = 3.14, CI = 1.50-13.3, p=0.002, Q-value not calculated).

### 3.5 HLA-Class I and II associations in sporadic pediatric LGG and HGG

We also identified HLA alleles that were significantly associated in the sporadic pediatric LGG cohort. More specifically HLA-B*13:02 (OR = 2.18, CI = 1.32-3.43, p=0.002, Q = 0.049) and HLA-DRB1*16:01 (OR = 3.93, CI = 2.16-6.63, p=1.6x10^-5^, Q = 0) were highly significant. HLA-DRB1*16:01 also had a nominal p-value of 0.01 in sporadic pediatric HGG, but the false discovery was not calculated as this allele did not reach our 5% frequency threshold in observed or expected alleles to be considered. Similarly HLA-B*13:02 had a nominal p-value of 0.038 in NF1 associated HGG, but did not pass our false discovery cutoff (Q = 0.586), possibly due to the limited size of the NF1 HGG cohort investigated.

## 4 Discussion

In this study, we have identified several HLA alleles that are associated with a higher rate of central and peripheral nervous system tumor formation in NF1 individuals, including NF1-HGG, NF1-LGG or MPNST. Although our analyses reviewed retrospective data in an exploratory manner, the findings we identified are statistically robust and should be seen as hypothesis-generating associations that warrant further investigation. Primarily, these associations merit independent prospective validation on larger cohorts of NF1 individuals to identify alleles that may yield a clinically actionable increase in risk for NF1 associated tumors. These findings should also prompt functional investigation to explore the mechanisms why specific HLA alleles or haplotypes are associated with NF1 tumor formation.

We speculate several hypotheses that warrant further studies: (1) *Infection susceptibility*: Specific alleles can predispose patients to viral or bacterial infections, which in turn can contribute to tumor formation. For example, associations between HLA-DRB1*04:03 and cytomegalovirus have been described (34). Additionally, specific HLA alleles enhance the risk for human papillomavirus persistence and initiation of cervical cancer (35). However, as far as we are aware, no viral infections have been associated with tumor formation in NF1. (2) *Ability of HLA-class I alleles to display tumor specific peptides*: The primary function of HLA class I receptors is to present peptides to which the immune system can respond. Alleles associated with tumor formation could have a reduced ability to display specific tumor peptides that trigger the immune system. However, one would expect that the other HLA alleles would compensate for this deficiency, making this hypothesis of how HLA alleles are associated with tumor formation less likely. In contrast, underrepresentation of HLA alleles in patients with cancer (i.e., protection against a tumor), can be explained by the efficient presentation/binding of a tumor-specific peptides/neoantigens in the grove of the HLA receptor to elicit a T-cell response. (3) *Drivers of tumor formation closely linked to HLA-genes*: A polymorphism or gene variant closely linked to the associated HLA haplotype could predispose to tumor formation in NF1. Several genes, other than HLA genes, are present in the MHC genomic locus where all HLA class I genes reside. One of them, *POU5F1* (*OCT4*), is of interest as it is an important transcription factor with a role in cancer stem cells (36). *POU5F1* is located within 290kb from HLA-B and C, and 1.2Mb from HLA-A. This close physical location indicates they could be in linkage disequilibrium.

HLA-class II receptors are typically only presented by antigen presenting immune cells; however, recent data shows that a substantial number of NF1-HGG tumor cells express the HLA-class II antigen presenting machinery (17). Similar observations were made in other cancers, although the percentage of tumor cells that express the antigen presentation machinery is substantially lower than what was observed in NF1-HGG (37–39). The specific mechanism of how HLA-class II expression on tumor cells contributes to tumor formation remains elusive, but it is suggested they play an immune suppressive role through stimulation of CD4 T-cells and stimulation of exhaustion of CD8 T-cells. It also remains unclear whether the presentation of specific antigens in HLA-class II molecules is important. Specific HLA-class II alleles can thus have a tumor promoting or protecting role through expression on immune cells or tumor cells.

It is known that HLA alleles are associated with or underrepresented in patients with cancer. In the general population the actual lifetime risk for cancer when you carry such an associated allele usually remains low, as the observed ORs are often in the 1–3 range and the baseline lifetime risk for a specific cancer itself is very low. However, in patients that have an elevated baseline risk to develop tumors, like individuals with NF1, this modest change in OR can dramatically affect outcomes. The association of HLA alleles with tumor formation in predisposition syndromes like NF1 can thus have a direct impact on patient care. Particularly for MPNST, the odds ratios of the observed associations suggest a dramatic increase in the likelihood of an NF1 patient developing malignancies. HLA-B*40:02 could increase the lifetime risk for MPNST to 16.1-57.2%, compared to the baseline risk of 10-15.8% in the general NF1 population. Although this risk elevation reaches statistical significance, the broad confidence interval reflects the limited sample size of allele carriers in the cohort. Moreover, this lifetime risk estimate model is based on the assumptions of the most recent retrospective analysis, which is expected to be revised over time. Consequently, while the potential magnitude of risk enrichment is compelling, larger cohorts are required to refine the precision of this estimate before its clinical significance can be determined. The HLA data reported here warrant further research into HLA genotype-phenotype associations in Neurofibromatosis type 1. Of note, further studies are expected to expand the HLA typing to all known HLA class II alleles to more comprehensively investigate these associations. This will require more extensive targeted HLA typing of NF1 individuals and prospective validation to define clinically actionable alleles. Taken together, this report is a call to action for the community to consider HLA-typing of NF1 individuals and to validate these observations in a large HLA genotype-phenotype study, with the hope that one day HLA data may improve the clinical care of those with NF1.

## Data Availability

The datasets presented in this study can be found in online repositories. The names of the repository/repositories and accession number(s) can be found in the article/**Supplementary Material**.
